# Prophylactic Mastectomy: Postoperative Skin Flap Thickness Evaluated by MRT, Ultrasound and Clinical Examination

**DOI:** 10.1245/s10434-019-08157-2

**Published:** 2020-01-06

**Authors:** Rebecca Wiberg, Magnus N. Andersson, Johan Svensson, Anna Rosén, Freja Koch, Annika Björkgren, Malin Sund

**Affiliations:** 1 Department of Surgical and Perioperative Sciences, Plastic Surgery and Surgery, Umeå University, Umeå, Sweden; 2 Department of Statistics, Umeå School of Business, Economics and Statistics, Umeå University, Umeå, Sweden; 3 Department of Radiation Sciences, Oncology and Radiology, Umeå University, Umeå, Sweden

## Abstract

**Background:**

Women with an increased hereditary risk of breast cancer can undergo prophylactic mastectomy (PM), which provides a significant, but not total, risk reduction. There is an ongoing discussion about how much skin and subcutaneous tissue should be resected to perform an adequate PM while leaving viable skin flaps.

**Methods:**

Forty-five women who had undergone PM were examined with magnetic resonance tomography (MRT), ultrasound (US) and clinical examination (CE) by a plastic surgeon and a general surgeon to estimate skin flap thickness.

**Results:**

The estimated mean skin flap thickness after PM was 13.3 (± 9.6), 7.0 (± 3.3), 6.9 (± 2.8) and 7.4 (± 2.8) mm following MRT, US, and CE performed by a plastic surgeon and a general surgeon, respectively. The mean difference in estimated skin flap thickness was significant between MRT and the other measuring methods, while there was no significant difference between US and CE, nor between CE performed by the surgeons. The mean skin flap thickness was significantly affected by the age at PM. Following PM, necrosis was detected in 7/23 (30.4%) of the breasts in skin flaps ≤ 5 mm and in 5/46 (10.9%) of the breasts in skin flaps > 5 mm (OR 6.29; CI 1.20–32.94; *p* = 0.03).

**Conclusion:**

The odds of getting postoperative necrosis was > 6 times higher in skin flaps ≤ 5 mm. Thus, if the degree of remaining glandular tissue is acceptably low, it is desirable to create skin flaps thicker than 5 mm to prevent wound healing problems after the PM procedure.

**Electronic supplementary material:**

The online version of this article (10.1245/s10434-019-08157-2) contains supplementary material, which is available to authorized users.

## Introduction

Breast cancer is the most common type of cancer among women, with approximately every ninth woman being affected before the age of 75 in Sweden.^1^ Five to ten percent of all breast cancers are due to hereditary factors,^1^ but only a minority of all hereditary breast cancers are mediated by pathogenic variants in highly penetrant genes.^2^ Pathogenic variants in the breast cancer genes BRCA1/2 account for 2–5% of all breast cancers,^1^ resulting in a 50–80% lifetime risk of breast cancer and a 10–60% lifetime risk of ovarian cancer.^3^^,^^4^ Because of the high lifetime risk of breast cancer, women with identified pathogenic variants in BRCA1/2 are recommended annual screening. In addition, these women are informed about the possibility of undergoing risk-reducing prophylactic mastectomy (PM; removal of the breasts) and prophylactic bilateral salpingo-oophorectomy (PBSO; removal of ovaries and fallopian tubes).^1^ The oncological risk associated with residual mammary tissue is not fully clear, but PM is estimated to reduce breast cancer risk by about 90%,^5^ with an even greater risk reduction seen in women who have also undergone PBSO (95% risk reduction).^6^

To date, little is known about the prevalence and localization of residual breast tissue after a mastectomy. Glandular tissue increases with thicker skin flaps, with skin flaps > 5 mm showing significantly more residual glandular tissue.^7^ However, skin flap necrosis is reported in 30% of the patients following mastectomy with skin flaps 4–5 mm,^8^ in comparison to 5% skin flap necrosis for all types of mastectomies.^9^

The local recurrence rate is around 5% following mastectomy after cancer, with skin sparing mastectomy having equivalent oncological outcomes to simple mastectomy.^10^ In Sweden, following mastectomy due to cancer, women are offered annual surveillance with breast imaging, after which they return to the population-based screening program consisting of mammography every 18th to 24th month until the age of 74. After PM, breast cancer is diagnosed in approximately 1–1.9% of all women.^5^ In contrast to women that undergo mastectomy due to cancer, there is no published consensus or guidelines regarding appropriate medical follow-up for those who opt for risk reducing surgery.

The aim of this study was to compare estimated skin flap thickness with different imaging modalities and CE and to explore the correlation between skin flap thickness and risk for necrosis.

## Methods

Women with increased hereditary risk of breast cancer that had undergone PM at the Department of Plastic surgery at Umeå University Hospital between 1997 and 2016 were invited to the study. Inclusion criteria were PM with or without implant-based reconstruction. Exclusion criteria were mastectomy due to cancer and reconstruction with autologous tissue. Out of an eligible population of 73 women, 27 women choose not to participate and one woman was excluded due to very high BMI, preventing MRT. Each breast was analyzed at four locations corresponding to the middle of the inner upper quadrant, inner lower quadrant, outer lower quadrant and outer upper quadrant, rendering a total of 284 possible measuring points per analysis, with the number of examined quadrants stated for each analysis.

Interpretation of the MRT and US images was performed by two different breast radiologists, performing approximately half of the interpretations each, and the CEs were performed by two plastic surgeons, dividing the patients among them, and two general surgeons, also dividing the patients among them. Each patient underwent CE by both a plastic surgeon and a general surgeon. CE included palpation but also measuring skin flap thickness using a caliper. Thus, in this context, skin flap thickness represents the skin’s thickness measured at follow-up examinations, rather than the preoperative thickness of the skin flaps. The skin flap thickness represents the entire skin flap, including epidermis, dermis, subcutaneous fat and possible glandular tissue. Both CE and imaging were performed at the same time point.

The quadrants of the left and right breast were examined in regard to thickness of the skin flap and any suspicion of tumors, after which the findings were documented in pre-printed clinical reporting forms in a standardized manner. The mean skin flap thickness per breast was calculated by adding the skin flap thickness for each quadrant and dividing it by four. The skin flap mean (SD) was measured in mm.

All relevant medical records were reviewed. Data that were difficult to obtain from medical records were collected by a health questionnaire given to the patients on the day of examination or sent to them by mail. Patient data were registered from the date of their PM until end of follow-up (9 December 2017). Complications after PM were recorded as follows; hematoma defined as hematoma leading to surgical evacuation; infection defined as clinically suspected infection leading to antibiotic treatment; necrosis defined as all kind of necrosis including partial skin necrosis/epidermolysis to full thick necrosis; and implant loss regardless of etiology.

### Statistics

Patient characteristics and frequencies of events were summarized using descriptive statistical methods. To assess the difference in estimated skin flap thickness between different breast quadrants and between different measuring methods, a general linear model (GLM) repeated measure design was used with within subject factors of breast quadrants (four levels), breast location (two levels), and measuring method (four levels). The Bonferroni method was used as a post hoc test when assessing the difference between skin flap thickness between breast quadrants within each measurement method. Furthermore, the difference in estimated skin flap thickness between different measuring methods was also analyzed using Bland–Altman plots. A generalized estimating equation (GEE) model was used to analyze how different factors affected the skin flap thickness mean. A GEE-model with binary logistic link was also used to analyze how different factors affected the degree of necrosis. In both GEE models an exchangeable covariance structure was used. Data analyses were performed by SPSS statistical software (IBM SPSS 22). Test results with *P* value of less than 0.05 were considered statistically significant.

### Ethics

The study was performed according to the principles of the Declaration of Helsinki and ethical guidelines of the Swedish Research Council. Ethical approval was obtained from the regional vetting board in Umeå (Dnr 2017-141-31M, 20170530). All participating patients gave informed written consent.

## Results

### Study Population

Forty-five women met the inclusion criteria, rendering 71 PM breasts with or without implant-based reconstruction to be included in the analysis (Supplementary Fig. 1). Out of these 45 women, five did not undergo MRT and two did not undergo US (Supplementary Fig. 2); one woman did not undergo MRT due to claustrophobia (A25), one woman due to implants containing metal (A28), two women refused MRT as they recently had breast MRT at another hospital, and were unwilling to undergo the same procedure again (A35 and A36), and finally, one woman did not undergo MRT because she had too high BMI to fit into the MRT machine (A40). Regarding the breast MRT performed at another hospital (A35 and A36), the protocol differed too much from that performed at our unit to allow us to include these images in the present study. Ultrasound was not performed in one woman because 2 weeks previously she had undergone surgery due to a spontaneous hematoma of the breast with implant removal and hematoma evacuation, making it difficult to interpret the results (A28 sin), and one woman did not undergo US due to an implant rupture, also complicating the interpretation of the imaging (A29).

Mean age at PM was 43.3 (± 10.4) years and mean time between PM and follow up was 8.0 (± 5.5) years. Of these 45 women, 21 underwent bilateral PM without a previous cancer, 15 underwent contralateral PM and 9 underwent bilateral PM after a previous cancer operated on using breast conserving surgery. Thus, the term “prophylactic mastectomy” is used both when there is no previous history of breast cancer in the breast, as well as in the setting of PM after a previous operation with breast conserving surgery due to cancer. Of all women, 53.3% (24/45) had a history of previous breast cancer, while 46.7% (21/45) had PM on both sides, corresponding to 7.0% (5/71) breasts with cancer and 93.0% (66/71) breasts without a previous cancer. Following PM, no women got breast cancer in the ipsilateral breast. Clinical characteristics and treatment modalities for all patients are presented in Table 1.

**Table 1:** Clinical characteristics and surgical history; clinical characteristics and surgical history in 45 women and 71 breasts

	%
Age at prophylactic mastectomy, years	
Mean (SD)	43.3 (± 10.4)
Time between prophylactic mastectomy and follow up, years	
Mean (SD)	8.0 (± 5.5)
Surgical history	
Prophylactic bilateral mastectomy without previous cancer	46.7 (21/45)
Prophylactic contralateral mastectomy	33.3 (15/45)
Prophylactic bilateral mastectomy due to previous cancer	
Operated with breast conserving therapy	20.0 (9/45)
Women with previous breast cancer	
Yes	53.3 (24/45)
No	46.7 (21/45)
Breasts with previous cancer	
Yes	7.0 (5/71)
No	93.0 (66/71)
Women with cancer in ipsilateral breast after prophylactic mastectomy	
Yes	0
No	100 (45/45)
Smoking	
Yes	8.9 (4/45)
No	91.1 (41/45)
Hypertension	
Yes	13.3 (6/45)
No	86.7 (39/45)
Diabetes	
Yes	0
No	100 (45/45)
BMI preoperatively	
Mean (SD)	25.0 (± 3.9)
BMI at follow up	
Mean (SD)	26.1 (± 4.3)

### Skin flap Thickness

The mean skin flap thickness was calculated by adding the skin flap thickness from each quadrant and dividing it by the total number of quadrants. The skin flap thickness was investigated with MRT (*n* = 248 quadrants), US (*n* = 276 quadrants) and CE. The CE was performed by both a plastic surgeon (*n* = 284 quadrants) and a general surgeon (*n* = 284 quadrants). The mean skin flap thickness after PM was estimated to be 13.3 (± 9.6) mm by MRT, 7.0 (± 3.3) mm by US and 6.9 (± 2.8) and 7.4 (± 2.8) mm following CE performed by a plastic surgeon and a general surgeon, respectively.

To assess difference in skin flap thickness between different breast quadrants a GLM repeated measure design was used with within subject factors of breast quadrants (four levels) and breast location (two levels). The skin flap thickness differed significantly between the different breast quadrants (Table 2). However, there was not just one quadrant that significantly differed from the others, but pronounced differences were observed within several of the quadrants. No significant difference was observed between the left and right breast (*p* = 0.447).

**Table 2:** Skin flap thickness in different quadrants

	Q1	Q2	Q3	Q4	*p* values
	Mean (CI)	Mean (CI)	Mean (CI)	Mean (CI)	
MRT (*n* = 22 women)	12.2 (8.4–16.0)	13.6 (9.9–17.2)	10.4 (7.4–13.3)	11.7 (8.8–14.5)	0.003 (Q2 vs Q3)
US (*n* = 24 women)	6.6 (5.5–7.6)	7.6 (6.1–9.1)	6.1 (4.8–7.4)	5.3 (4.4–6.1)	0.011 (Q1 vs Q4) 0.000 (Q2 vs Q3) 0.000 (Q2 vs Q4)
CE plastic surgeon (*n* = 26 women)	6.4 (5.3–7.4)	7.2 (6.0–8.3)	6.9 (5.7–8.0)	5.9 (5.0–6.8)	
CE general surgeon (*n* = 26 women)	8.0 (7.0–9.0)	7.0 (6.2–7.7)	6.4 (5.7–7.1)	7.2 (6.2–8.2)	0.007 (Q1 vs Q3)

The skin flap thickness (mm) in the upper inner quadrant (Q1), lower inner quadrant (Q2), lower outer quadrant (Q3) and upper outer quadrant (Q4) following prophylactic mastectomy was investigated with magnetic resonance tomography (MRT) (*n* = 22 women), ultrasound (US) (*n* = 24 women) and clinical examination (CE) performed by a plastic surgeon (*n* = 26 women) and a general surgeon (*n* = 26 women). Differences in estimated skin flap thickness between the different quadrants were calculated by a general linear model repeated measure design. *p* values are for difference in pair-wise means. *p* values are adjusted with Bonferroni method

The difference in estimated skin flap thickness between different measuring methods was analyzed with a GLM repeated measure design with within subject factors of breast quadrants (four levels), breast location (two levels) and measuring methods (four levels) (*n* = 21 patients) and through Bland–Altman plots. The mean difference in estimated skin flap thickness was significantly different between MRT and US (5.40; CI 1.72–9.08; *p* = 0.002), MRT and CE performed by a plastic surgeon (5.36; CI 1.63–9.09; *p* = 0.003) and MRT and CE performed by a general surgeon (4.82; CI 1.01–8.62; *p* = 0.008), while there was no significant difference between US and CE performed by a plastic surgeon (*p* = 1.0) nor a general surgeon (*p* = 1.0). Neither was there a significant difference between CE performed by a plastic surgeon and a general surgeon (*p* = 0.65). Through the Bland–Altman plots we determined the mean difference between the scores and the limits of agreement (two SD from the mean difference). A similar pattern was observed on the Bland–Altman plots as with the GLM repeated measure design, where the mean difference was pronounced when MRT was compared against US and CE, while the difference was very small between US and CE and between CE performed by a plastic surgeon and a general surgeon (Supplementary Fig. 3).

### Factors Associated with Skin Flap Thickness Measured by Ultrasound

A GEE model was used to analyze how different factors affected the mean skin flap thickness measured with US following PM (*n* = 68 breasts). Since the estimated skin flap thickness differed significantly between MRT and US, while there was no significant difference between US and CE, the analysis was performed only on skin flap thickness measured with US. With adjusted analysis, the mean skin flap thickness was significantly affected by the age at prophylactic surgery, with thicker skin flaps observed with increasing age (0.104 mm/year; CI 0.029–0.178; *p* = 0.006) while BMI preoperatively and BMI change between surgery and follow-up did not affect the skin flap thickness significantly (Table 3).

**Table 3:** Factors associated with skin flap thickness

	Unadjusted analysis	Adjusted analysis
	B-coefficient	B-coefficient
Age at prophylactic surgery, mm per year	0.129 (CI 0.049–0.209; *p *= 0.002)	0.104 (C1 0.029–0.178; *p *= 0.006)
BMI preoperatively, mm per kg/m^2^	0.286 (0.040–0.533; *p *= 0.023)	0.140 (C1 –0.084–0.364; *p *= 0.222)
BMI change between surgery and follow-up, mm per kg/m^2^	− 0.185 (–0.445–0.075; *p *= 0.164)	− 0.173 (CI –0.451–0.105; *p *= 0.934)

A generalized estimating equation model was used to analyze how different factors affected the skin flap thickness mean measured with ultrasound following prophylactic mastectomy (*n* = 68 breasts). The observed effect on the skin flap thickness mean was expressed with B-coefficient and confidence interval (CI). In the adjusted analyses all variables to the left were included

### Complications Following Prophylactic Mastectomies

Complications after PM were evaluated in 71 breasts. The most common complication was skin necrosis, reported in 16.9% (12/71) of all breasts. Wound infection, hematoma and implant loss was reported in 8.5% (6/71), 7.0% (5/71) and 7.0% (5/71) of all breasts, respectively.

### Factors Associated with Necrosis

Correlation between necrosis and skin flaps ≤ 5 mm (*n* = 23), previous radiotherapy (*n* = 5) and smoking (*n* = 6) was calculated with a GEE-model with binary logistic link. Due to the low number of breasts with necrosis, only an unadjusted analysis was performed. Mean skin flap thickness per breast measured with US and necrosis was investigated following PM in two different groups; skin flaps ≤ 5 mm (*n* = 23 breasts) and skin flaps > 5 mm (*n* = 46 breasts). Following PM, necrosis was detected in 30.4% (7/23) of the breasts in skin flaps ≤ 5 mm and in 10.9% (5/46) of the breasts in skin flaps > 5 mm. Both skin flap thickness ≤ 5 mm (OR 6.29; CI 1.20–32.94; *p* = 0.03) and previous radiotherapy (OR 21.97; CI 2.49–193.81; *p* = 0.005) were predictors for necrosis following PM, in contrast to smoking that did not significantly affect the degree of necrosis (OR 0.95; CI 0.12–7.29; *p* = 0.958) (Table 4).

**Table 4:** Factors associated with necrosis

	OR
Skin flaps ≤ 5 mm	6.29 (CI 1.20–32.94; *p *= 0.03)
Previous radiotherapy	21.97 (CI 2.49–193.81; *p *= 0.005)
Smoking	0.95 (CI 0.12–7.29; *p *= 0.958)

Correlation between necrosis and skin flaps ≤ 5 mm (*n* = 23 of 69 breasts), preoperative radiation (*n* = 5 of 71 breasts), and smoking (*n* = 6 of 71 breasts) was calculated with an unadjusted generalized estimating equation model with binary logistic link. The observed effect of necrosis was expressed with odds ratio (OR) and confidence interval (CI)

## Discussion

To our knowledge, skin flap thickness following mastectomy has not been evaluated with imaging before. Neither has the correlation between skin flap thickness and necrosis been addressed in this manner.

PM is a surgical procedure that has increased dramatically over recent years, and not only among women with increased hereditary risk of breast cancer. Kummerow et al. 11 demonstrated that bilateral mastectomy as a treatment for unilateral breast cancer increased from 1.9% in 1998 to 11.2% in 2011 in the United States. Moreover, Hawley et al.^12^ showed that 69% of all women who underwent contralateral PM had no genetic or familial risk factors. Complications are common after skin sparing mastectomy, with necrosis seen in up to 30–40% of patients, 8^,^^13^^,^^14^ and approximately 10% need subsequent surgical intervention.^13^ This highlights the need to evaluate the residual oncological risk versus the risk of surgical complication after PM.

There is an ongoing discussion about how much skin and subcutaneous tissue should be resected to perform an adequate mastectomy while leaving viable skin flaps.^15^ The ideal would be to create skin flaps that are thin enough to remove all breast tissue but at the same time are thick enough to preserve flap circulation. One common recommendation is to dissect just superficial to the superficial fascia of the breast.^15^ Beer et al.^16^ demonstrated that the superficial fascial layer was absent in 44% of breast resection specimens, and even when present and visible, the distance to the overlying skin was usually very small, < 1.1 mm in 50% of patients. Larson et al.^17^ identified a distinct layer of non-breast-bearing subcutaneous tissue between the dermis and the breast parenchyma suggesting that oncologically safe flaps can be at least 1 cm thick, since they have shown that the median thickness of dermis is 0.57 cm and the non-breast bearing subcutaneous thickness has a median thickness of 10 mm.

We demonstrate here that the mean skin flap thickness after PM was estimated to be 13.3 (± 9.6) mm measured by MRT, 7.0 (± 3.3) mm by US and 6.9 (± 2.8) and 7.4 (± 2.8) mm following CE performed by a plastic surgeon and a general surgeon, respectively. Furthermore, we show that there was a significant mean difference in estimated skin flap thickness between MRT and US (*p* = 0.002), MRT and CE performed by a plastic surgeon (*p* = 0.003) and MRT and CE performed by a general surgeon (*p* = 0.008), while there was no significant difference between US and CE (*p* = 1.0), nor between CE performed by a plastic surgeon and a general surgeon (*p* = 0.65). A similar pattern was observed using the Bland–Altman plots (Supplementary Fig. 3). Thus, US and CE seem to be comparable methods for estimating skin flap thickness, while MRT might overestimate the thickness. The thicker skin flap measured with MRT can in part be explained by the position of the patient. MRT is performed with the patient lying face down and breasts hanging freely. In contrast, during US, the patient is lying on her back, allowing the breast tissue to “spread out.” Also, pressure from the probe could make skin appear even thinner on US.

There are few studies regarding the location of residual glandular tissue in the breasts, and all seem to be based on histological preparations.^18^^,^^19^ Griepsma et al. 18 investigated > 7000 biopsy samples from 206 patients after a mastectomy, and showed that residual breast tissue was found with a significant predilection for the lower outer quadrant and the middle circle of the superficial dissection plane. We demonstrate that the skin flap thickness differed significantly between the different breast quadrants (Table 2), while no significant difference was observed between the left and right breasts (*p* = 0.447). However, there was not just one quadrant that significantly differed from the others, but pronounced differences were observed within several of the quadrants.

We showed that the skin flap thickness mean was significantly affected by the age at prophylactic surgery, with thicker skin flaps observed with increasing age (*p* = 0.006) (Table 3). According to the literature, the subcutaneous tissue thickness can be extremely variable and does not seem to correlate with BMI or patient age. 16^,^^17^ The increased awareness around the correlation between increased skin flap thickness and remaining glandular tissue, might, in part, explain why thinner skin flaps were observed in younger women.

Furthermore, we demonstrate that skin flap necrosis was the most common complication following PM, reported in 18.9% of all breasts. The risk of skin flap necrosis increased significantly with thinner skin flaps, with necrosis detected in 30.4% of the breasts with skin flaps ≤ 5 mm and in 10.9% of those with skin flaps > 5 mm. Moreover, both skin flap thickness ≤ 5 mm (*p* = 0.03) and preoperative radiotherapy (*p* = 0.005) were predictors for necrosis following PM, in contrast to smoking that did not significantly affect the degree of necrosis in the present cohort (*p* = 0.958) (Table 4). However, caution should be taken when interpreting the results, since the number of smokers and women treated with preoperative radiotherapy was very low.

Although achieving oncological safety is the primary aim with PM, too thin skin flaps affects the circulation, with decreased skin flap viability as a result, potentially delaying systemic oncologic treatment and compromising the esthetic outcome. The overall incidence of mastectomy skin flap necrosis is around 5%,^9^ while skin flap necrosis of varying degrees is reported in 30% of patients following mastectomy with skin flaps 4–5 mm.^8^ Arver et al.^14^ showed that following bilateral PM, partial skin necrosis/epidermolysis was the most common complication and reported in 30% of the patients. This usually involved only a part of the preserved areola or the skin edges, while necrosis leading to implant loss was a rare complication. Cao et al.^20^ analyzed the correlation between remaining glandular tissue and skin flap thickness in 168 skin sparing mastectomies. A biopsy was taken from the skin flap overlying the tumor, with glandular tissue found more often in thicker biopsies (12 mm compared with 8.6 mm, *p* = 0.019), supporting the hypothesis that the risk of remaining glandular tissue is increased with skin flaps thicker than 10 mm. Furthermore, Torresan et al.^7^ stated that skin flaps > 5 mm significantly increases the risk of residual glandular tissue. Thus, the degree of necrosis increases with thinner skin flaps, with 5-mm-thick skin flaps reported in the literature to be associated with a high degree of necrosis,^8^ while, in contrast, the amount of remaining glandular tissue seems to increase significantly with skin flaps > 5–10 mm.^8^^,^^20^

Our study has limitations. Twenty-seven women chose not to participate for unknown reasons, mediating risk of selection bias. Furthermore, it would have been desirable to have all imaging reviewed by two different radiologists, to be able to calculate correlation between examiners. Although the number of measuring points was high, there were few participants in the study.

Each breast was analyzed at four locations corresponding to the middle of each quadrant. However, since MRT is performed with the patient lying face down and breasts hanging freely, in contrast to US, when the patient is lying on her back, allowing the breast tissue to “spread out”, the “middle of each quadrant” might differ somewhat between US and MRT.

In conclusion, the odds of getting necrosis is more than 6 times higher in skin flaps ≤ 5 mm after PM. Thus, if the degree of residual glandular tissue is acceptably low, it is desirable to create skin flaps thicker than 5 mm to prevent wound healing problems. More research is needed to establish the relationship between skin flap thickness and residual glandular tissue, as well as the oncological impact of residual glandular tissue, since clearer surgical guidelines are desired to reduce the high amount of necrosis associated with PM. US and CE seem to be better methods for measuring skin flap thickness, since the position of the patient during MRT may overestimate the measure of skin flap thickness.

## Electronic supplementary material

Below is the link to the electronic supplementary material.

Flow chart of inclusions and exclusions (TIFF 2702 kb)

Overview of the surgical history and the surveillance modalities for all women. The following codes are used for the surgical history: prophylactic bilateral mastectomy (PBM) without previous cancer = 1, prophylactic contralateral mastectomy = 2 and PBM due to previous cancer operated with breast conserving therapy = 3. The following abbreviations are used for the reconstruction: local fasciocutaneous flap = LFF, latissimus dorsi flap = LD, deep inferior epigastric perforator flap = DIEP, and no reconstruction = NR. Women highlighted in gray are included in the analysis (DOCX 23 kb)

Bland–Altman plot for inter-observer agreement of skin flap thickness. The skin flap thickness (mm) was estimated with magnetic resonance tomography (MRT), ultrasound (US) and clinical examination (CE) performed by a plastic surgeon and a general surgeon. The Bland–Altman plot shows the difference between the two skin flap thickness scores plotted against the average of the two skin flap thickness scores for the following comparisons: **A** Inter-observer agreement between MRT and US (n = 244 quadrants). Mean difference between scores (6.16) and limits of agreement (− 10.13 to 22.45). **B** MRT and CE performed by a plastic surgeon (n = 248 quadrants). Mean difference between scores (6.33) and limits of agreement (− 10.13 to 22.79). **C** MRT and CE performed by a general surgeon (n = 248 quadrants). Mean difference between scores (5.91) and limits of agreement (− 10.85 to 22.67). **D** US and CE performed by a plastic surgeon (n = 276 quadrants). Mean difference between scores (0.007) and limits of agreement (− 4.91 to 4.93). **E** US and CE performed by a general surgeon (n = 276 quadrants). Mean difference between scores (0.39) and limits of agreement (− 6.18 to 6.96). **F** CE performed by a plastic surgeon and a general surgeon (*n* = 284 quadrants). Mean difference between scores (0.44) and limits of agreement (− 5.42 to 6.3). The *bold line* indicates the mean difference between scores and the *dotted lines* show the limits of agreement (TIFF 1366 kb)
