# Supplementary figures and images for: Prophylactic Mastectomy: Postoperative Skin Flap Thickness Evaluated by MRT, Ultrasound and Clinical Examination
Source: Ann Surg Oncol. 2020 Jan 6;27(7):2221–8. doi: 10.1245/s10434-019-08157-2 (PMC7311506; doi:10.1245/s10434-019-08157-2)

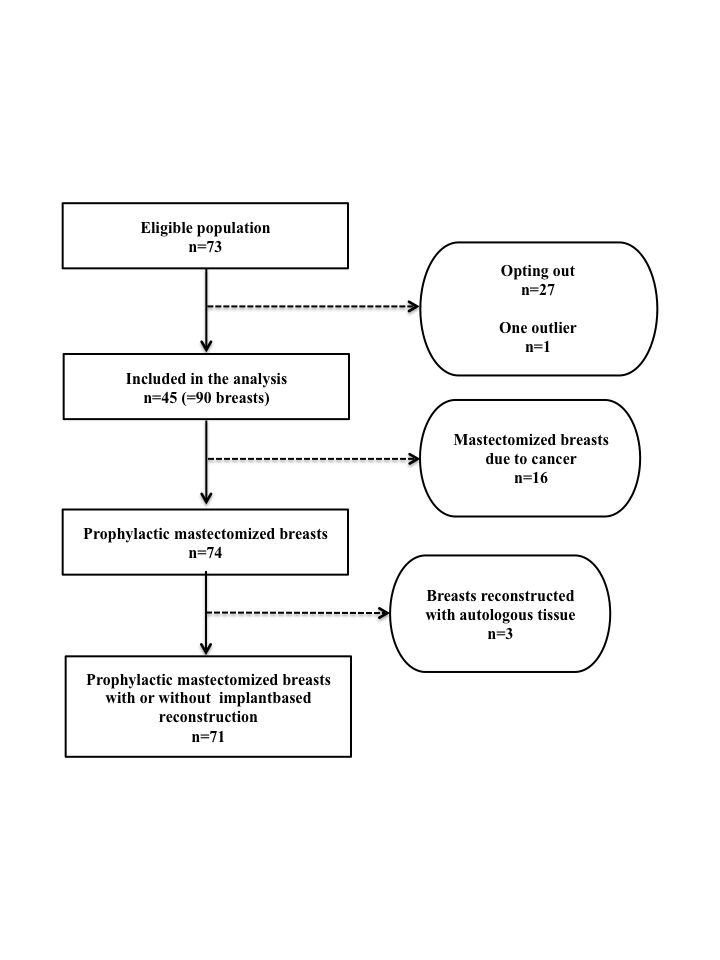

Supplement: Supplementary file 1 — Flow chart of inclusions and exclusions (TIFF 2702 kb) [file 10434_2019_8157_MOESM1_ESM.tiff]

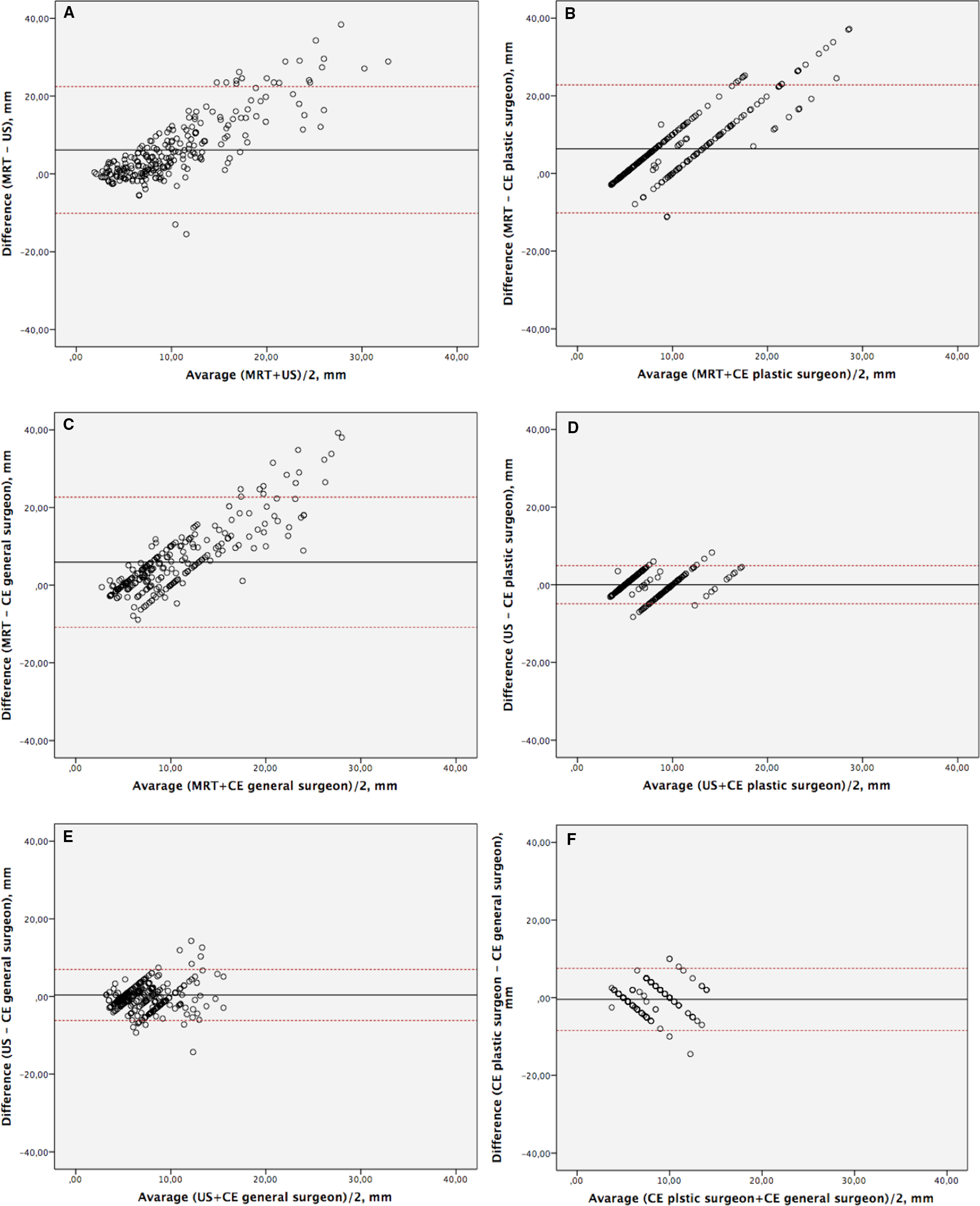

Supplement: Supplementary file 3 — Bland–Altman plot for inter-observer agreement of skin flap thickness. The skin flap thickness (mm) was estimated with magnetic resonance tomography (MRT), ultrasound (US) and clinical examination (CE) performed by a plastic surgeon and a general surgeon. The Bland–Altman plot shows the difference between the two skin flap thickness scores plotted against the average of the two skin flap thickness scores for the following comparisons: A Inter-observer agreement between MRT and US (n = 244 quadrants). Mean difference between scores (6.16) and limits of agreement (− 10.13 to 22.45). B MRT and CE performed by a plastic surgeon (n = 248 quadrants). Mean difference between scores (6.33) and limits of agreement (− 10.13 to 22.79). C MRT and CE performed by a general surgeon (n = 248 quadrants). Mean difference between scores (5.91) and limits of agreement (− 10.85 to 22.67). D US and CE performed by a plastic surgeon (n = 276 quadrants). Mean difference between scores (0.007) and limits of agreement (− 4.91 to 4.93). E US and CE performed by a general surgeon (n = 276 quadrants). Mean difference between scores (0.39) and limits of agreement (− 6.18 to 6.96). F CE performed by a plastic surgeon and a general surgeon (n = 284 quadrants). Mean difference between scores (0.44) and limits of agreement (− 5.42 to 6.3). The bold line indicates the mean difference between scores and the dotted lines show the limits of agreement (TIFF 1366 kb) [file 10434_2019_8157_MOESM3_ESM.tiff]
