# Supplementary material for: Prophylactic Mastectomy: Postoperative Skin Flap Thickness Evaluated by MRT, Ultrasound and Clinical Examination
Source: Ann Surg Oncol. 2020 Jan 6;27(7):2221–8. doi: 10.1245/s10434-019-08157-2 (PMC7311506; doi:10.1245/s10434-019-08157-2)
Supplement: Supplementary file 2 — Overview of the surgical history and the surveillance modalities for all women. The following codes are used for the surgical history: prophylactic bilateral mastectomy (PBM) without previous cancer = 1, prophylactic contralateral mastectomy = 2 and PBM due to previous cancer operated with breast conserving therapy = 3. The following abbreviations are used for the reconstruction: local fasciocutaneous flap = LFF, latissimus dorsi flap = LD, deep inferior epigastric perforator flap = DIEP, and no reconstruction = NR. Women highlighted in gray are included in the analysis (DOCX 23 kb) [file 10434_2019_8157_MOESM2_ESM.docx]

|  |  |  |  |  |  |  |  |  |  |  |  |
| --- | --- | --- | --- | --- | --- | --- | --- | --- | --- | --- | --- |
|  | **Case No** | **Surgical** | **Breast** | **Previous** | **Prophylactic** | **Reconstruction** | **MRT** | **US** | **CE** | **CE** |  |
|  |  | **history** |  | **cancer** | **surgery** |  |  |  | **plastic** | **general** |  |
|  | A1 | 3 | sin | no | yes | Implant | yes | yes | yes | yes |  |
|  | A1 | 3 | dx | yes | yes | implant | yes | yes | yes | yes |  |
|  | A2 | 1 | sin | no | yes | implant | yes | yes | yes | yes |  |
|  | A2 | 1 | dx | no | yes | implant | yes | yes | yes | yes |  |
|  | A3 | 1 | sin | no | yes | implant | yes | yes | yes | yes |  |
|  | A3 | 1 | dx | no | yes | implant | yes | yes | yes | yes |  |
|  | A4 | 3 | sin | yes | yes | DIEP | yes | yes | **no** | yes |  |
|  | A4 | 3 | dx | no | yes | implant | yes | yes | yes | yes |  |
|  | A5 | 1 | sin | no | yes | implant | yes | yes | yes | yes |  |
|  | A5 | 1 | dx | no | yes | implant | yes | yes | yes | yes |  |
|  | A6 | 3 | sin | yes | yes | implant | yes | yes | yes | yes |  |
|  | A6 | 3 | dx | no | yes | implant | yes | yes | yes | yes |  |
|  | A7 | 1 | sin | no | yes | implant | yes | yes | yes | yes |  |
|  | A7 | 1 | dx | no | yes | implant | yes | yes | yes | yes |  |
|  | A8 | 1 | sin | no | yes | implant | yes | yes | yes | yes |  |
|  | A8 | 1 | dx | no | yes | implant | yes | yes | yes | yes |  |
|  | A9 | 3 | sin | no | yes | implant | yes | yes | yes | yes |  |
|  | A9 | 3 | dx | yes | yes | implant | yes | yes | yes | yes |  |
|  | A10 | 1 | sin | no | yes | implant | yes | yes | yes | yes |  |
|  | A10 | 1 | dx | no | yes | implant | yes | yes | yes | yes |  |
|  | A11 | 2 | sin | no | yes | implant | yes | yes | yes | yes |  |
|  | A11 | 2 | dx | yes | no | Implant + LFF | yes | yes | yes | yes |  |
|  | A12 | 2 | sin | no | yes | implant | yes | yes | yes | yes |  |
|  | A12 | 2 | dx | yes | no | implant | yes | yes | yes | yes |  |
|  | A13 | 2 | sin | no | yes | implant | yes | yes | yes | yes |  |
|  | A13 | 2 | dx | yes | no | implant | yes | yes | yes | yes |  |
|  | A14 | 2 | sin | no | yes | implant | yes | yes | yes | yes |  |
|  | A14 | 2 | dx | yes | no | implant | yes | yes | yes | yes |  |
|  | A15 | 2 | sin | no | yes | implant | yes | yes | yes | yes |  |
|  | A15 | 2 | dx | yes | no | implant + LD | yes | yes | yes | yes |  |
|  | A16 | 2 | sin | no | yes | implant | yes | yes | yes | yes |  |
|  | A16 | 2 | dx | yes | no | implant + LD | yes | yes | yes | yes |  |
|  | A17 | 2 | sin | no | yes | no reconstruction | yes | yes | yes | yes |  |
|  | A17 | 2 | dx | yes | no | no reconstruction | yes | yes | yes | yes |  |
|  | A18 | 2 | sin | yes | no | no reconstruction | yes | yes | yes | yes |  |
|  | A18 | 2 | dx | no | yes | no reconstruction | yes | yes | yes | yes |  |
|  | A19 | 3 | sin | yes | no | implant + LD | yes | yes | yes | yes |  |
|  | A19 | 3 | dx | no | yes | implant | yes | yes | yes | yes |  |
|  | A20 | 2 | sin | yes | no | implant | yes | yes | yes | yes |  |
|  | A20 | 2 | dx | no | yes | implant | yes | yes | yes | yes |  |
|  | A21 | 2 | sin | yes | no | implant | yes | yes | yes | yes |  |
|  | A21 | 2 | dx | no | yes | implant | yes | yes | yes | yes |  |
|  | A22 | 2 | sin | yes | no | implant | yes | yes | yes | yes |  |
|  | A22 | 2 | dx | no | yes | implant | yes | yes | yes | yes |  |
|  | A23 | 2 | sin | yes | no | implant + LFF | yes | yes | yes | yes |  |
|  | A23 | 2 | dx | no | yes | implant | yes | yes | yes | yes |  |
|  | A24 | 1 | sin | no | yes | implant | yes | yes | yes | yes |  |
|  | A24 | 1 | dx | no | yes | implant | yes | yes | yes | yes |  |
|  | A25 | 3 | sin | yes | yes | implant + LD | **no** | yes | yes | yes |  |
|  | A25 | 3 | dx | no | yes | implant | **no** | yes | yes | yes |  |
|  | A26 | 3 | sin | no | yes | implant | yes | yes | yes | yes |  |
|  | A26 | 3 | dx | yes | yes | implant + LD | yes | yes | yes | yes |  |
|  | A27 | 1 | sin | no | yes | implant | yes | yes | yes | yes |  |
|  | A27 | 1 | dx | no | yes | implant | yes | yes | yes | yes |  |
|  | A28 | 1 | sin | no | yes | implant | **no** | **no** | yes | yes |  |
|  | A28 | 1 | dx | no | yes | implant | **no** | yes | yes | yes |  |
|  | A29 | 1 | sin | no | yes | implant | yes | **no** | yes | yes |  |
|  | A29 | 1 | dx | no | yes | implant | yes | yes | yes | yes |  |
|  | A30 | 1 | sin | no | yes | implant | yes | yes | yes | yes |  |
|  | A30 | 1 | dx | no | yes | implant | yes | yes | yes | yes |  |
|  | A31 | 3 | sin | yes | yes | implant | yes | yes | yes | yes |  |
|  | A31 | 3 | dx | no | yes | implant | yes | yes | yes | yes |  |
|  | A32 | 1 | sin | no | yes | implant | **no** | yes | yes | yes |  |
|  | A32 | 1 | dx | no | yes | implant | **no** | yes | yes | yes |  |
|  | A33 | 1 | sin | no | yes | implant | yes | yes | yes | yes |  |
|  | A33 | 1 | dx | no | yes | implant | yes | yes | yes | yes |  |
|  | A34 | 1 | sin | no | yes | implant | yes | yes | yes | yes |  |
|  | A34 | 1 | dx | no | yes | implant | yes | yes | yes | yes |  |
|  | A35 | 1 | sin | no | yes | implant | **no** | yes | yes | yes |  |
|  | A35 | 1 | dx | no | yes | implant | **no** | yes | yes | yes |  |
|  | A36 | 1 | sin | no | yes | implant | **no** | yes | yes | yes |  |
|  | A36 | 1 | dx | no | yes | implant | **no** | yes | yes | yes |  |
|  | A37 | 3 | sin | yes | yes | implant | yes | yes | yes | yes |  |
|  | A37 | 3 | dx | no | yes | implant | yes | yes | yes | yes |  |
|  | A38 | 2 | sin | no | yes | implant | yes | yes | yes | yes |  |
|  | A38 | 2 | dx | yes | no | implant + LFF | yes | yes | yes | yes |  |
|  | A39 | 1 | sin | no | yes | implant | yes | yes | yes | yes |  |
|  | A39 | 1 | dx | no | yes | implant | yes | yes | yes | yes |  |
|  | A40 | 1 | sin | no | yes | implant | **no** | yes | yes | yes |  |
|  | A40 | 1 | dx | no | yes | implant | **no** | yes | yes | yes |  |
|  | A41 | 1 | sin | no | yes | implant | yes | yes | yes | yes |  |
|  | A41 | 1 | dx | no | yes | implant | yes | yes | yes | yes |  |
|  | A42 | 1 | sin | no | yes | implant | yes | yes | yes | yes |  |
|  | A42 | 1 | dx | no | yes | implant | yes | yes | yes | yes |  |
|  | A43 | 2 | sin | no | yes | implant | yes | yes | yes | yes |  |
|  | A43 | 2 | dx | no | no | implant | yes | yes | yes | yes |  |
|  | A44 | 1 | sin | no | yes | implant | yes | yes | yes | yes |  |
|  | A44 | 1 | dx | no | yes | implant | yes | yes | yes | yes |  |
|  | A45 | 2 | sin | no | yes | implant | yes | yes | yes | yes |  |
|  | A45 | 2 | dx | yes | no | implant + LD | yes | yes | yes | yes |  |
|  | A46 | 1 | Sin | no | yes | implant | yes | yes | yes | yes |  |
|  | A46 | 1 | dx | no | yes | implant | yes | yes | yes | yes |  |
|  | Surgical history: 1 = Prophylactic bilateral mastectomy (PBM) without previous cancer, 2 = Prophylactic contralateral mastectomy, | | | | | | | | | |  |
|  | 3 = PBM due to previous cancer operated with breast conserving therapy | | | | | |  |  |  |  |  |
|  | Reconstruction: LD = latissimus dorsi flap, DIEP = Deep inferior epigastric perforator flap, NR = No reconstruction | | | | | | | | |  |  |
|  |  |  |  |  |  |  |  |  |  |  |  |
